# CLUSTERING OF MULTIDIMENSIONAL NEIGHBORHOOD CHARACTERISTICS IN SHAPING HEALTHY AGING

**DOI:** 10.1093/geroni/igae098.2123

**Published:** 2024-12-31

**Authors:** Jiao Yu, Weidi Qin, Jiuzhou Wang, Eva Kahana

**Affiliations:** Yale University, New Haven, Connecticut, United States; University of Wisconsin Madison, Madison, Wisconsin, United States; University of Minnesota, Minneapolis, Minnesota, United States; Case Western Reserve University, Cleveland, Ohio, United States

## Abstract

Neighborhoods are pivotal in shaping later life health and well-being. While previous research has primarily examined a single dimension of neighborhood risk factors (e.g., socioeconomic disadvantages), this study proposes a timely shift toward a multi-dimensional perspective of neighborhood characteristics. We aim to explore how multiple neighborhood characteristics synergistically shape various later life health outcomes, including comorbidity, disabilities (ADL), mental health, functional limitations, cognitive health, and epigenetic aging (GrimAge). Using data from the 2014 and 2016 waves of the Health and Retirement Study linked to the National Neighborhood Data Archive (n = 8,474), we first applied a machine learning algorithm -- the Partitioning Around Medoids (PAM) method to identify a neighborhood typology based on neighborhood-level socioeconomic status, facilities, services, and hazards. This analysis identified four clusters: affluent neighborhoods, mid-SES with high hazards neighborhoods, high amenity neighborhoods, and disadvantaged neighborhoods. Subsequently, we conducted multilevel regression analyses to examine the association between the identified neighborhood clusters and later life health outcomes. Regression results revealed significant health disparities across different neighborhood clusters. Compared to their counterparts living in affluent neighborhoods, older adults residing in disadvantaged neighborhoods and those in mid-SES with high hazards neighborhoods experienced greater chronic conditions, worse mental health, and more functional limitations. These findings underscore the urgent need for tailored interventions aimed at enhancing service facilities and mitigating environmental hazards in disadvantaged and high-risk communities. Addressing these neighborhood factors is essential for promoting communities that are conducive to healthy aging and supportive of successful aging in place.

